# Nucleopore Traffic Is Hindered by SARS-CoV-2 ORF6 Protein to Efficiently Suppress IFN-β and IL-6 Secretion

**DOI:** 10.3390/v14061273

**Published:** 2022-06-11

**Authors:** Gianni Gori Savellini, Gabriele Anichini, Claudia Gandolfo, Maria Grazia Cusi

**Affiliations:** Department of Medical Biotechnologies, University of Siena, 53100 Siena, Italy; gabriele.anichini@student.unisi.it (G.A.); claudia.gandolfo@unisi.it (C.G.); mariagrazia.cusi@unisi.it (M.G.C.)

**Keywords:** interleukin-6, SARS-CoV-2 ORF6 protein, interferon antagonist

## Abstract

A weak production of INF-β along with an exacerbated release of pro-inflammatory cytokines have been reported during infection by the novel SARS-CoV-2 virus. SARS-CoV-2 encodes several proteins that are able to counteract the host immune system, which is believed to be one of the most important features contributing to the viral pathogenesis and development of a severe clinical outcomes. Previous reports demonstrated that the SARS-CoV-2 ORF6 protein strongly suppresses INF-β production by hindering the RIG-I, MDA-5, and MAVS signaling cascade. In the present study, we better characterized the mechanism by which the SARS-CoV-2 ORF6 counteracts IFN-β and interleukin-6 (IL-6), which plays a crucial role in the inflammation process associated with the viral infection. In the present study, we demonstrated that the SARS-CoV-2 ORF6 protein has evolved an alternative mechanism to guarantee host IFN-β and IL-6 suppression, in addition to the transcriptional control exerted on the genes. Indeed, a block in movement through the nucleopore of newly synthetized messenger RNA encoding the immune-modulatory cytokines IFN-β and IL-6 are reported here. The ORF6 accessory protein of SARS-CoV-2 displays a multifunctional activity and may represent one of the most important virulence factors. Where conventional antagonistic strategies of immune evasion—such as the suppression of specific transcription factors (e.g., IRF-3, STAT-1/2)—would not be sufficient, the SARS-CoV-2 ORF6 protein is the trump card for the virus, also blocking the movement of IFN-β and IL-6 mRNAs from nucleus to cytoplasm. Conversely, we showed that nuclear translocation of the NF-κB transcription factor is not affected by the ORF6 protein, although inhibition of its cytoplasmic activation occurred. Therefore, the ORF6 protein exerts a 360-degree inhibition of the antiviral response by blocking as many critical points as possible.

## 1. Introduction

Coronaviruses (CoVs) are a large family of single-stranded, positive-sense RNA viruses that belong to the family Coronaviridae [1]. Since the end of 2019, the outbreak of a novel coronavirus Severe Acute Respiratory Syndrome coronavirus 2 (SARS-CoV-2) has emerged, which then rapidly spread worldwide until the World Health Organization (WHO) declared a pandemic in March 2020 [2,3]. SARS-CoV-2 causes COronaVIrus Disease 2019 (COVID-19), which includes a variable spectrum of symptoms, ranging from mild influenza-like syndrome to severe pneumonia, acute respiratory distress, and even death [4,5,6,7]. Whereas SARS-CoV and MERS-CoV have tropism for lower airways, the emergent SARS-CoV-2 virus is present at a high viral load in the upper respiratory tract [8,9]. Although the SARS-CoV-2 genome structure follows that of known CoVs, based on the current annotation it encodes six accessory proteins atypical to the viral family (3a, 6, 7a, 7b, 8, and 10) [10]. Early virus-mediated immune suppression is believed to be one of the most important characteristics of SARS-CoV-2 infection and could contribute, at least in part, to the viral pathogenesis. Indeed, the suppression or delay of type I interferons (IFN-α/β) expression is associated with the COVID-19 disease [11]. The type I IFN-mediated immune response represents the first line of host defense against viral infection due to the engagement of pattern recognition receptors (PRRs), including retinoic-acid inducible gene-I (RIG-I), melanoma differentiation-associated protein 5 (MDA-5), and toll-like receptors (TLRs) [12,13]. The activation of PRRs triggers a signaling cascade which leads to the activation of transcription factors (interferon regulatory factor 3/7, IRF-3/7; nuclear factor kappa-light-chain-enhancer of activated B cells, NF-κB; activator protein 1, AP-1) involved in type I IFNs expression and the further establishment of an antiviral state [12,13]. Therefore, a tight control of the signaling cascade mediated by the PRRs, which leads to the final production of IFN-α/β, is required [14].

Some authors suggested that the onset of severe lung injury in SARS-CoV-2 infected patients depends on the induction of a cellular stress that is coupled with the activation of specific transcription factors, such as NF-κB, that results in an exacerbated proinflammatory host response. Many lines of evidence have suggested that the overproduction of inflammatory cytokines plays a major role in the pathogenesis of SARS-CoV-2 infection, as well as in the progression and severity of the respiratory disease [15,16,17]. Many viral proteins have been associated with the activation of the mitogen-activated protein kinase (MAPK)/NF-*κ*B pathway, controlling the expression of apoptotic and pro-inflammatory related genes (IL-6, TNF-α, and IL-1β) [18,19,20]. The most important key pathogenic proteins of SARS-CoV-2 are represented by the nucleoprotein N [21,22,23,24] and the non-structural proteins NSP1, NSP3, NSP12 and NSP14 [25,26]. In addition to the above-mentioned viral proteins, the ORF6 has been involved in viral innate immunity evasion and with the alteration of molecules trafficking through the nucleopore complex (NPC) [25,26,27,28,29,30].

In the present study, we better investigated the role of SARS-CoV-2 ORF6 protein on both IFN-β and inflammatory cytokines expression, such as interleukin-6 (IL-6). We described the antagonistic behavior of the SARS-CoV-2 ORF6 protein using luciferase reporter gene assay and unconventional data interpretation to demonstrate its function. Indeed—and conversely to that observed by others—the expression of the internal control represented by the *Renilla* luciferase, was drastically decreased at protein level by the presence of ORF6 [25,31]. Furthermore, reporter gene assay demonstrated that the over-expression of SARS-CoV-2 ORF6 protein negatively affected the expression of the reporter gene downstream the full-length IFN-β promoter. Conversely, reporter gene expression controlled by the IL-6 promoter was not affected by the presence of the viral protein, which correlated to the unperturbed NF-κB nuclear translocation herein described. A similar activity was observed towards both endogenous cytokines at transcriptional level, reporting a downregulation of IFN-β, while a marginal, although significant, inhibition of IL-6 transcription occurred. Notwithstanding, both IL-6 and IFN-β proteins were not secreted by ORF6 expressing cells, suggesting that, although the transcriptional inhibitory function of the ORF6 is evident with respect to IFN-β, an alternative mechanism is in control of IL-6 expression.

Since the viral protein interacts with the nucleopore protein complex (NPC), we hypothesized that the antagonistic nature of the ORF6 on certain cellular proteins could be—at least in part—due to limited movement of specific mRNAs from nucleus to cytoplasm; in particular, those encoding proteins involved in innate immunity or inflammation. Lee et al. suggested that ORF6 might hamper host mRNA export mechanisms to further suppress the immune response in order to favor the host translation machinery for viral replication [29]. In agreement with these results, we also reported a marked retention of IFN-β and IL-6 mRNAs in the cell nucleus, consistent with the relative protein deregulation. Furthermore, we demonstrated that the modulation of IFN-b and IL-6 exerted by SARS-CoV-2 ORF6 was strikingly influenced by domains located at the C-terminus of the protein. Indeed, it has been previously reported that the M58R ORF6 mutant is deficient in binding Rae1 and Nup98 nucleopore components due to alteration of a diacidic motif located at the end of the ORF6 (aa 53–56) [27,28,32].

These findings suggest that the SARS-CoV-2 ORF6 protein represent an important virulence factor, controlling—at multiple steps and with different strategies—the host immune and inflammatory response, thus genetic variability occurring on the accessory viral protein should be considered.

## 2. Aim of the Study

The study aims to further characterize SARS-CoV-2 ORF6 protein function towards the pro-inflammatory cytokine interferon-beta (IFN-β) and interleukin-6 (IL-6). From the point of view of innate immunity antagonism, the viral protein has been well characterized. Indeed, it has been demonstrated that ORF6 affinity to the nucleopore complex proteins (NPC) blocks IRF-3 and STAT-1/2 nuclear translocation, leading to the suppression of type I IFN production and signaling. On the contrary, the impact of the viral protein on IL-6 production, which is highly increased during infection, has not been investigated before. Thus, ORF6 regulatory properties on IL-6 expression were investigated. Furthermore, we also analyzed whether NPC hindrance had consequences regarding innate immunity evasion. Moreover, the involvement of ORF6 protein C-terminal domain on both IFN-β and IL-6 production was investigated, concluding that the terminal part of the protein is crucial for its function.

## 3. Materials and Methods

### 3.1. Cells, Virus, and Chemicals

Human embryonic kidney HEK-293T cells (Merck Millipore, Milan, Italy) and A549 (ATCC CCL-185) were cultured in Dulbecco’s modified Eagle’s medium (DMEM) (Lonza, Milan, Italy) supplemented with 100 U/mL penicillin/streptomycin (Hyclone Europe, Milan, Italy) and 10% heat-inactivated fetal calf serum (FCS) (Lonza) at 37 °C. SARS-CoV-2 was isolated on Vero E6 cells (ATCC CRL-1586) from a clinical specimen at the Virology laboratory of “S. Maria alle Scotte” Hospital in Siena, Italy. Transfections were performed using the jetPRIME Transfection Reagent (Polyplus, Milan, Italy) following the manufacturer’s instructions. The NF-κB inhibitors *Resveratrol* and the polyinosinic–polycytidylic acid (poly(I:C)) were purchased from Merck Millipore.

### 3.2. Plasmids

The HA-tagged SARS-CoV-2 wild-type (USA-WA1/2020; BEI Resources NR-52281) and M58R ORF6 variants expressing plasmids were kindly provided by Prof. A. García-Sastre (CEIRS program; NIAD Centers of Excellence for Influenza Research and Surveillance; Mount Sinai School of Medicine, New York, NY, USA). The reporter plasmid encoding firefly luciferase downstream of the complete (pIFN-β) or the minimal (pIFN-κB) interferon-beta promoter were a gift by Prof. Takashi Fujita (Tokyo Metropolitan Institute of Medical Science, Tokyo, Japan). Luciferase reporter plasmids containing the full (pIL-6pIL-6) or the NF-κB minimal IL-6 promoter (pIL-6-κB) were kindly provided by Prof. M. Palmieri (university of Verona, Verona, Italy), the IL10 promoter-based reporter plasmid was a gift by Prof. C. Hedrich (University of Liverpool, Liverpool, UK), while the Inhibitor of nuclear factor kappa-B kinase subunit alpha (IKK-α) was a kind gift of Prof. Ludwig (University of Münster, Münster, Germany).

### 3.3. Immunoblotting

Cell pellets were lysed in standard RIPA buffer (50 mM Tris-HCl (pH7.5), 150 mM NaCl, 1 mM EDTA, 1% Triton X-100) supplemented with anti-proteases cocktail (Roche, Milan, Italy) and anti-phosphatase cocktails 2 and 3 (Merck Millipore). Protein concentration was determined by Bradford assay (Pierce, Milan, Italy). Fifty µg of total proteins were prepared in 1× Laemmli sample buffer (50 mM Tris-HCl (pH 6.8), 2% SDS, 10% (w/v) glycerol, 4% β-Mercaptoethanol, 0.05% bromophenol blue), denatured for 5 min by boiling, and separated by SDS-PAGE. Resolved proteins were transferred to nitrocellulose membrane (Santa Cruz Biotechnology, Heidelberg, Germany) and, after blocking with 5% non-fat dry milk, the filters were incubated with anti-HA (Merck Millipore) (1:2000) or anti-actin (BioLegend, London, UK) as a loading control overnight (o/n) at room temperature (RT). After being washed three times with PBS-T (phosphate buffered saline, 0.05% Tween-20), membranes were incubated with horseradish peroxidase (HRP)-conjugated anti-mouse IgG secondary antibody (Merck Millipore) for 1 h at RT. Immunocomplexes were revealed using the TMB-Blotting 1-Step Solution (Pierce). Reported figures were representative of at least three independent experiments.

### 3.4. Luciferase Reporter Assay

HEK-293T or A549 cells (2 × 10^5^/well) were seeded in 24-well plates and, after o/n incubation at 37 °C, the cells were transfected with 200 ng of the complete (pIFN-β) or minimal interferon-beta promoter (pIFN-κB), the full-length (pIL-6) or the NF-κB minimal IL-6 promoter (pIL-6-κB) luciferase reporter plasmids, alone or in combination with indicated amounts of ORF6 or its M58R mutant expressing plasmids. Where indicated, 200 ng of inhibitory-κB kinase alpha (IKK-α) expressing plasmid was co-transfected. Transfections were performed by using the jetPRIME Transfection Reagent (Polyplus) following the manufacturer’s instruction. Total transfected DNA amount was kept constant by using empty plasmid. As internal control, pSV40-RL was co-transfected in order to monitor transfection efficiency and cell viability. Samples were collected at 24 h or 48 h post-transfection and luciferase activities (relative light units; RLU) were assessed on cell lysates by the dual-luciferase reporter assay reagent (Promega, Milan, Italy).

Poly(I:C) stimulation was achieved at 36 h post-transfection by transfecting 1 μg/mL of poly(I:C) using jetPRIME transfection reagent. After an additional 12 h, cells were collected and luciferase activities (RLU) were measured.

To assess the effects of NF-κB inhibitor on pIFN-κB and pIL-6 promoters’ activation, transfected cells were treated with 10 μM of vehicle (DMSO) or *Resveratrol* for 12 h. Samples were collected at 48 h post-transfection and luciferase values were measured. Results were reported as mean firefly or *Renilla* RLU values of at least three independent experiments ± standard deviations (SD).

### 3.5. Cell Viability Assay

The cytotoxic effects induced by the ORF6 expression on cell cultures was determined by the CyQUANT MTT Cell Viability Assay (Invitrogen, Milan, Italy). Cultures of HEK-293T cells were plated in 24-well at 2 × 10^5^ cells/well. The day after, cells were transfected with increasing amounts (10 ng and 500 ng) of wild-type ORF6 or M58R mutant expressing plasmids, with 500 ng of green fluorescent protein (GFP) expressing plasmid or 500 ng of empty vector by using jetPRIME transfection reagent (Polyplus) following manufacturer’s instructions. The MTT assay was assessed accordingly to manufacturer’s instruction. Briefly, at 48 h post-transfection, cell monolayers were incubated for 4 h at 37 °C with (3-(4,5-dimethylthiazol-2-yl)-2,5-diphenyltetrazolium bromide (MTT) diluted in complete culture medium without phenol red to a final concentration of 1.2 mM. Formazan insoluble precipitate was then solubilized with dimethyl sulfoxide (DMSO) for 10 min and then optical density (O.D.) values were recorded at 540 nm. Cell viability was calculated as percentage of the fold changes between experimental samples and control sample. Data were plotted as mean cell viability percentage values ± standard deviations (SD). Significance was determined using unpaired, two-tailed Student’s *t*-test (**** *p* < 0.00001, *** *p* < 0.0005, ** *p* < 0.005, * *p* < 0.05, n.s. = not significant).

### 3.6. Cellular Fractionation

To test NF-κB nuclear translocation, sub-cellular fractionation was performed. Empty vector (Ctr-), ORF6 pr M58R mutant transfected cells were collected at 48 h post-transfection and lysed in buffer A (250 mM sucrose, 20 mM HEPES (pH 7.4), 10 mM KCl, 1.5 mM MgCl_2_, 1 mM EDTA, 1 mM EGTA, 1 mM DTT) supplemented with complete protease inhibitor cocktail (Roche) and anti-phosphatases cocktails 2 and 3 (Merck Millipore). After 30 min of incubation on ice, samples were centrifuged at 12,000× *g* for 30 min and supernatant fractions were stored as cytoplasmic soluble proteins. The crude nuclear fractions (pellets) were washed twice with buffer A, centrifuged ad described above, and the resulting pellets were resuspended in solution B (50 mM Tris HCl (pH 8), 150 mM NaCl, 0.5% sodium deoxycholate, 1% NP-40, 0.1% SDS), incubated in the cold (4 °C) for 30 min with gentle agitation and then centrifuged at 12,000× *g* for 10 min. After centrifugation, supernatant containing the nuclear proteins were collected and stored at −80 °C until used. Cytoplasmic and nuclear fractions protein content was achieved by standard Bradford reagent assay (Pierce) and 25 μg of proteins loaded for SDS–PAGE. Western blotting was performed by using anti-p65 NF-κB rabbit polyclonal antibody (ActiveMotif, Milan, Italy), anti-actin (BioLegend) or anti-Lamin B1 (Santa Cruz Biotechnologies) o/n at RT. As secondary antibodies, HRP-conjugated anti-rabbit IgG (Promega) or anti-mouse IgG (Merck Millipore) were used. Immunocomplexes were visualized by using 1-Step™ TMB-Blotting Substrate Solution (ThermoFisher Scientific, Milan, Italy) and quantitative comparison among samples was performed by densitometric analysis using ImageJ software.

### 3.7. Quantitative RT-qPCR

A549 cells (1 × 10^6^/well) were plated in 6-well plates and, after o/n incubation, cell monolayers were transfected with either 1 μg empty vector, wild-type, or M58R ORF6 variants or with green-fluorescent protein (GFP) expressing plasmids. Cells were collected at 24 h and 48 h post-transfection and total RNA was isolated using the RNeasy mini kit (Qiagen, Milan, Italy). For messenger (mRNA) sub-cellular distribution, transfected cells were subjected to cytoplasmic and nuclear fractionation, as previously described. Total RNA was purified from both cellular fractions using the RNeasy mini kit (Qiagen). The concentration of purified RNA samples was determined by spectrophotometric analysis with NanoDrop 2000 (Thermo Fisher Scientific, Milan, Italy). Equal amounts of RNA (100 ng) were used in reverse-transcription quantitative PCR (RT-qPCR) reactions with AgPath-ID™ One-Step RT-PCR Reagent (Thermo Fisher Scientific) in a QuantStudio 5 Real-Time PCR System (Thermo Fisher Scientific). TaqMan^®^ Assays (Thermo Fisher Scientific) for IFN-β, IL-6, and RNaseP were used for specific transcript detection. Each sample was run in duplicate and the cycle threshold (Ct) values of each gene were normalized against the endogenous RNaseP gene and compared with the negative, empty vector transfected, control. The results were represented as mean fold relative increments from at least three independent experiments (2^−^^ΔΔ^^Ct^ algorithm) ± standard deviations (SD). Significance was determined using unpaired, two-tailed Student’s *t*-test (**** *p* < 0.00001, *** *p* < 0.0005, ** *p* < 0.005, * *p* < 0.05, n.s. = not significant).

### 3.8. Cytokine Quantification by ELISA

Secretion of IL-6 and IFN-β by A549 cells was assessed on culture supernatant from ORF6-, wild-type or M58R mutant, or empty vector transfected A549 cells collected at both 24 h and 48 h post-transfection. Enzyme-linked immunosorbent assay (ELISA) was performed for IL-6 and IFN-β detection using the Human IL-6 Matched Antibody Pair kit (Thermo Fisher Scientific) or the VeriKine Human Interferon Beta ELISA Kit (PBL Assay Science**,** Piscataway, NJ, USA), respectively, following manufacturer’s instruction. Cytokine concentrations were expressed as pg/mL mean values from three independent experiments ± standard deviations (SD). Significance was determined using unpaired, two-tailed Student’s *t*-test (**** *p* < 0.00001, *** *p* < 0.0005, ** *p* < 0.005, * *p* < 0.05, n.s. = not significant).

### 3.9. Statistics

All data shown in graphs represent the mean of at least three independent experiments ± SD values. All statistical analyses were performed using GraphPad PRISM 6.0 software using one-way ANOVA or two-tailed unpaired Student’s *t*-test, as appropriate. *P*-values of *p* <  0.05 were considered to be statistically significant.

## 4. Results

### 4.1. Effects of SARS-CoV-2 ORF6 Protein on Cytokine Expression

**Figure 1:**
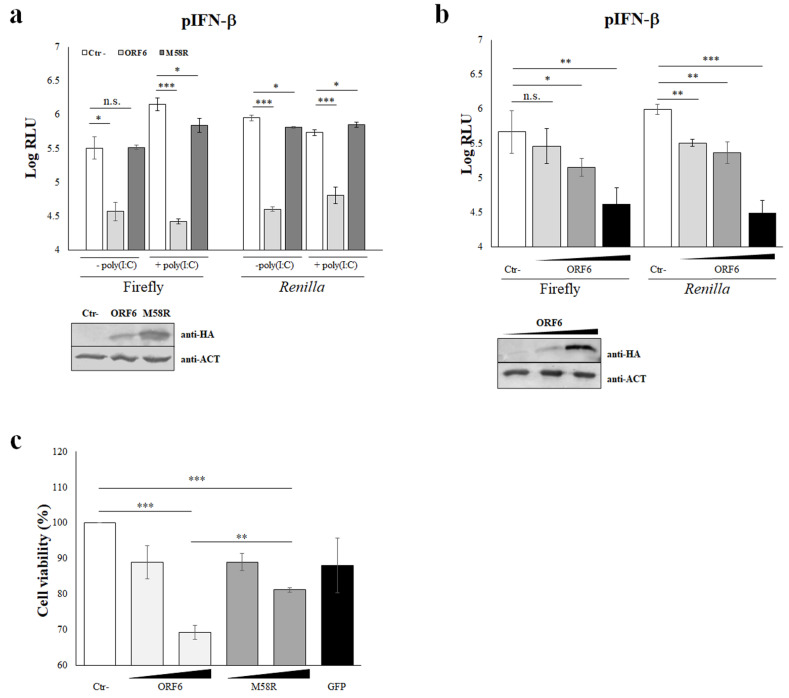
Inhibitory activity of SARS-COV-2 ORF6 protein. (**a**) HEK-293T cells were co-transfected with the IFN-β promoter-mediated firefly luciferase (pIFN-β) and the SV40 promoter-mediated *Renilla* luciferase reporter plasmids, with ORF6 or the mutant M58R expressing plasmids or with empty vector (Ctr-). At 36 h post-transfection, cells were left unstimulated or poly(I:C)-stimulated by transfection. Firefly and *Renilla* luciferase activities were estimated with respect to the relative control sample at 48 h post-transfection. Three (*n* = 3) independent experiments were performed and representative data are presented as mean logarithm values of relative luminescence unit (RLU) ± standard deviations (SD). Original blots are presented in Supplementary Materials. (**b**) Luciferase assay conducted in HEK-293T cells transfected with increasing ORF6 (wedge represents increasing amount of the expression vectors: 10, 100, and 500 ng) or empty vector (Ctr-), together with the pIFN-β and the SV40 promoter-mediated *Renilla* luciferase as the internal control. Data are presented as mean logarithm RLU values ± SD and are one representative of four independent experiments. (**c**) HEK-293T cells were co-transfected with increasing amounts (10 ng and 500 ng) of ORF6 or M58R expressing plasmids, with 500 ng of green fluorescent protein (GFP) expressing plasmid or with empty vector (Ctr-). Cell viability was evaluated at 48 h post-transfection by MTT assay. Three (*n* = 3) independent experiments were performed and representative data are presented as mean percentage of cell viability ± standard deviations (SD). (**a**–**c**) Significance was determined using unpaired, two-tailed Student’s *t*-test *** *p* < 0.0005, ** *p* < 0.005, * *p* < 0.05, n.s. = not significant).

The SARS-CoV-2 infection is responsible for uncontrolled pro-inflammatory cytokine production whereas a weak production of type I interferons (IFN-α/β) has been reported [25,26]. To study the role of the ORF6 protein of SARS-CoV-2 on IFN-α/β response, a reporter gene assay based on the firefly luciferase expression mediated by the IFN-β promoter (pIFN-β) was applied. Pattern recognition receptors (PRRs), such as RIG-I, play a crucial role in innate immunity as they mediate the IFN-β production following specific stimuli, such as viral infections or in vitro stimulation with the dsRNAs analogue, poly(I:C). Previous data showed that the ORF6 protein counteracts IFN-β production upon poly(I:C) stimulation by inhibiting RIG-I activation, thus IRF-3 phosphorylation and nuclear translocation [26]. In our study, we investigated the behavior of the wild-type (wt) ORF6 protein on the IFN-β system, in presence or absence of external stimuli. Moreover, the M58R ORF6 mutant—carrying a mutation in the C-terminal domain—was included as a control since it has been demonstrated to be inactive in antagonising IFN-β activity [27]. HEK-293T were co-transfected with the ORF6 genes along with the pIFN-β reporter plasmid and the internal control pSV40-RL, encoding the *Renilla* luciferase downstream the constitutively active SV40 promoter. At 48 h post-transfection, luciferase activities were measured in samples without external stimuli. Raw data of firefly luciferase values, expressed as resonance light units (RLU), demonstrated an inhibitory function for the wt-ORF6 protein, with an 8-fold reduction (*p* = 0.02; *) in IFN-β-specific promoter activation, while the M58R mutant did not display a significant antagonistic effect on the selected promoter activation (Figure 1a).

Subsequently, pIFN-β activation was evaluated after poly(I:C)-stimulation, confirming the marked antagonistic activity of the wt-ORF6 protein on IFN-β expression, leading to a 50-fold (*p* = 0.0001; ***) reduction in firefly reporter values (Figure 1a). On the contrary, the M58R ORF6 mutant showed reduced activity, since a partial inhibition of the IFN-β promoter activation was found (fold change 2, *p* = 0.02; *) (Figure 1a). Along with the specific promoter inhibition, the viral protein also exerted a very strong shutdown on internal control expression, *Renilla* luciferase. In fact, the expression of *Renilla* luciferase was also strongly decreased by the wt-ORF6 in unstimulated and poly(I:C)-stimulated samples by 22- (*p* = 0.0001, ***) and 8-fold (*p* = 0.0001; ***), respectively (Figure 1a). As a consequence, fold change (firefly/*Renilla* normalization) of reporter assay data led to a misinterpretation of ORF6 activity, thus single RLU were evaluated. Furthermore, the perturbation of the C-terminal part of the ORF6 evidenced the critical role of this domain in protein functions. Indeed, the M58R mutant almost completely lost activity towards *Renilla* expression, leading to a ~1.4- (*p* = 0.009; *) and ~0.8-fold (*p* = 0.02; *) reduction in reporter gene values in both unstimulated- and poly(I:C)-stimulated samples (Figure 1a). Furthermore, the reported function for the ORF6 protein was carried out in a dose-dependent manner, as demonstrated by transfecting increasing amounts of ORF6 expressing plasmid. Indeed, the IFN-β antagonistic property for the ORF6 was equally appreciated either at low (100 ng of plasmid DNA, 1.2-fold decrease, *p* = 0.015; *) or high (500 ng of plasmid DNA, 3-fold reduction, *p* = 0.0056; **) viral protein expression levels (Figure 1b). Similarly, the *Renilla* expression was inversely modulated by the ORF6 cellular levels. Surprisingly, when a lower expression of ORF6 was achieved by transfecting 10 ng plasmid DNA, a marginal, not significant, effect on IFN-β promoter activation was observed, although the *Renilla* downregulation was still appreciated (Figure 1b). To address whether the depicted ORF6 behavior was due to an artifact or intrinsic protein toxicity, we evaluated cell viability after low or high expression levels of both wt-ORF6 and M58R proteins. By MTT assay, a slight cytotoxic effect was observed when both protein variants were barely expressed (10 ng transfected plasmid), while they both induced cell damage with a significant reduction (1.4- and 1.2-fold, respectively) in cell viability when highly accumulated in the cells (Figure 1c). Notwithstanding, a marked difference in cellular viability between wt- (~1.5-fold decrease, *p* < 0.0001; ***) and mutated-ORF6 (~1.2-fold decrease, *p* = 0.001; **) proteins was noticed (Figure 1c). Control cells expressing high levels (500 ng of transfected plasmid) of green-fluorescent protein (GFP) displayed a marginal, not significant, effect on cell survival—similar to that observed when ORF6 proteins were barely expressed (Figure 1c).

### 4.2. ORF6 Protein Affects Transcriptional Activation of IL-6 and IL-10 Promoters

**Figure 2:**
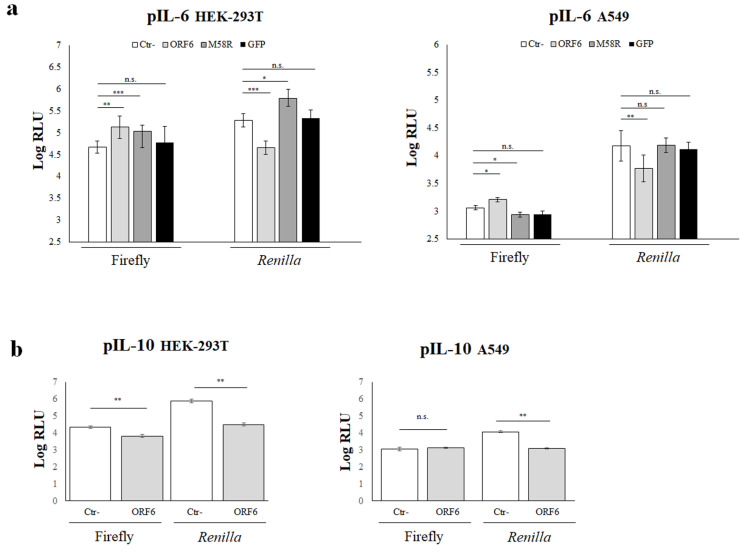
IL-6 and IL-10 promoter activation is deregulated by ORF6 protein. (**a**) HEK-293T (left panel) or A549 (right panel) cells were co-transfected with the interleukin-6 promoter-mediated firefly luciferase (pIL-6), along with the SV40 promoter-mediated *Renilla* luciferase reporter plasmids. Where indicated, ORF6, M58R mutant, or green-fluorescent protein (GFP) protein expression was achieved by transfection. Negative control (Ctr-) samples were transfected with empty plasmid along with reporter plasmids. At 48 h post transfection, firefly and *Renilla* luciferase activities were measured and compared to the relative control sample (Ctr-). Three (*n* = 3) independent experiments were performed and representative data are presented as mean logarithm values of relative luminescence unit (RLU) ± standard deviations. (**b**) The influence of ORF6 protein on pIL-10 activation was evaluated in both HEK-293T (left panel) and A549 (right panel) cells. Means of relative luminescence unit (RLU) logarithm values ± standard deviations are presented. (**a,b**) Significance was evaluated as *p* < 0.05 (*** *p* < 0.0005, ** *p* < 0.005, * *p* < 0.05, n.s. = not significant).

Unbalanced hyperproduction of pro-inflammatory cytokines has been observed in COVID-19 patients [11,19,20,21,22]. In the attempt to further characterize the ORF6 protein function, we investigated its effects on other important cellular mediators, interleukin-6 (IL-6) and interleukin-10 (IL-10). The pro-inflammatory IL-6 is the major cellular factor produced after cell damage induced by viral infection. It has been reported that IL-6 expression is controlled by the activity of several transcription factors with known consensus sequences in the IL-6 promoter region [15,16]. Similarly, a wide number of transcription factors have been characterized as essential or critical in IL-10 regulation. HEK-293T cells were transfected with wt- or M58R-ORF6 viral genes along with reporter plasmids containing the full-length IL-6- or IL-10-promoter (pIL-6, pIL-10) controlling the expression of the firefly luciferase. *Renilla* luciferase expression was achieved by transfection. When cytokine promoter activation was examined, both the wt- and mutated ORF6 proteins significantly (*p* = 0.0011, **; *p* = 0.0005, ***) upregulated the pIL-6 activity with a 2.8- and 2.5-fold increase, respectively, with respect to the negative control sample (Figure 2a).

The expression of the irrelevant GFP protein did not show any effect on pIL-6 promoter activation (Figure 2a), thus we concluded that the stimulatory function of the ORF6 is a peculiarity of the viral protein. On the contrary, the viral accessory protein wt-ORF6 retained its antagonistic function on pIL-10, with a 1.2-fold downregulation of its activity (*p* = 0.0016, **) (Figure 2b, left panel). Since HEK-293T cells do not represent the elective host for studying respiratory viruses, the pIL-6 and pIL-10 activation was further examined in another cell type. The human alveolar basal epithelial cells A549 were co-transfected with the pIL-6 or pIL-10-luciferase reporter, pSV40-RL internal control, and—where indicated—the ORF6 viral genes, empty vector, or GFP-expressing plasmid. At 48 h post-transfection, the reporter expression was examined. The new results matched with those obtained in HEK-293T cells, with a 1.4-fold stimulation on pIL-6 (*p* = 0.01, *) but not on pIL-10 (*p* = n.s.) by the wt-ORF6 (Figure 2a,b, right panels). The ORF6 M58R mutant partially lost its function as a slight reduction (~0.8-fold, *p* = 0.02, *) in the stimulation of pIL-6 was observed in the new cell line (Figure 2a, right panel). Based on these results, further experiments were conducted in HEK-293T cells, if not stated differently.

### 4.3. NF-κB Activity Is Not Affected by ORF6 of SARS-CoV-2

**Figure 3:**
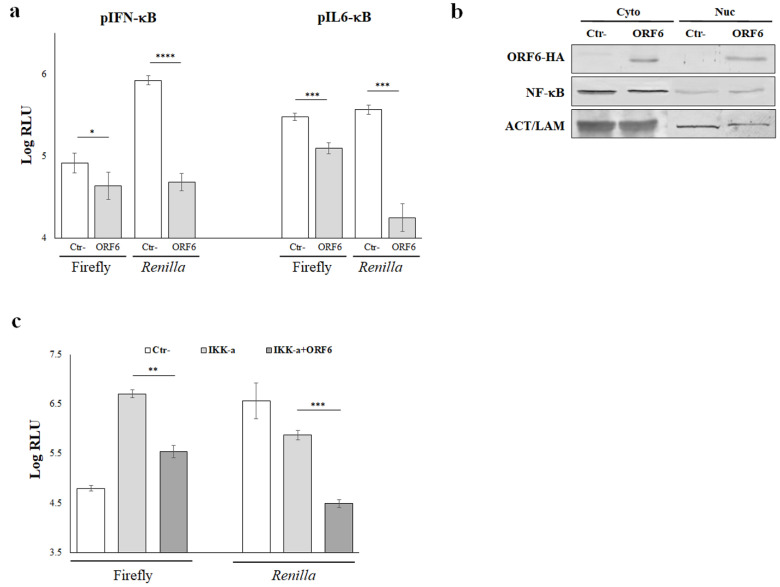
NF-κB activity is affected by ORF6 protein. (**a**) Interleukin-6 or -IFN-β minimal promoters containing the NF-kB responsive elements only (pIL6-κB and pIFNβ-κB, respectively) were used to drive firefly luciferase expression in HEK-293T cells. *Renilla* luciferase reporter plasmid was co-transfected as an internal control. Three (*n* = 3) independent measurements were performed and representative data are presented as mean logarithm values of relative luminescence unit (RLU) ± standard deviations. Significance was evaluated as *p* < 0.05 (**** *p* < 0.00001, *** *p* < 0.0005, ** *p* < 0.005, * *p* < 0.05, n.s. = not significant). (**b**) NF-κB nuclear translocation was evaluated in empty vector (Ctr-) or ORF6 expressing A549 cells. Cellular fractionation was performed as described in the Materials and Methods section and equal amounts of proteins (25 μg) were resolved by polyacrylamide gel. NF-κB movement into the nucleus was evaluated by probing the samples with anti-p65 NF-κB subunit specific antibody. In parallel, ORF6 subcellular localization was assessed in the same samples by anti-HA epitope tag antibody staining. Actin or lamin B1 were stained for cytoplasmic or nuclear loading control, respectively. Original blots are presented in Supplementary Materials. (**c**) Interleukin-6 promoter (pIL-6) was used to drive firefly luciferase expression in HEK-293T cells in combination with the ectopic expressing of the NF-κB activator IKK-α. Where indicated, empty plasmid (Ctr-) or the viral ORF6 protein expressing plasmid was co-transfected. *Renilla* luciferase reporter plasmid was used as internal control. Three (*n* = 3) independent measurements were performed and representative data are presented as mean logarithm values of relative luminescence unit (RLU) ± standard deviations. Significance was evaluated as *p* < 0.05 (**** *p* < 0.00001, *** *p* < 0.0005, ** *p* < 0.005, * *p* < 0.05, n.s. = not significant).

The expression and secretion of inflammatory cytokines in response to viral infections is a tightly controlled phenomenon in which NF-κB represents an important key regulatory element, working synergistically with other transcription factors. In the present study, we demonstrated that SARS-CoV-2 ORF6 protein displayed stimulatory properties on cellular promoters whose activity is strikingly influenced by the NF-κB transcription factor, such as that controlling IL-6 expression, while promoters whose activity is marginally influenced by NF-κB—such as those for INF-β and IL-10—were inhibited by the presence of ORF6 through different mechanisms. Thus, we examined whether NF-κB transcription factor was involved in the viral protein function. For this reason, we further evaluated the activation of IFN-β and IL-6 minimal promoters (pIFN-κB and pIL6-κB, respectively), consisting of the NF-κB responsive element only, in a luciferase reporter assay. HEK-293T cells were transfected with pIFN-κB or pIL6-κB and, where indicated, with wt-ORF6 viral gene. Forty-eight hours post-transfection, luciferase activities were measured and single reporter values were evaluated. ORF6 protein repressed the IFN-β minimal promoter pIFN-κB leading to a nearly 50% reduction in its activation (*p* = 0.026, *). Furthermore, pIL6-κB was also reduced (*p* = 0.0009, ***) by the ectopic expression of ORF6 with a 2.4-fold decrease in minimal promoter activation (Figure 3a).

These findings were further validated by examining the nuclear localization of the p65 NF-κB subunit. After cellular fractionation, the p65 nuclear translocation was tested by immunoblotting. Surprisingly, and contrary to the hindrance exerted by the wt-ORF6 with respect to other transcription factors, the wt-ORF6 protein did not hamper NF-κB movement to the nucleus, as the transcription factor was equally distributed in the cytoplasm and nuclear compartments (Figure 3b). Moreover, the wt-ORF6 protein was detected as part of the nuclear proteins, since its presence was detected in the nuclear fraction and, at a similar extent, in the cytoplasm (Figure 3b). Furthermore, we investigated the wt-ORF6 effects on pIL-6 activation by the inhibitory-κB kinase alpha (IKK-α), a NF-κB up-stream modulator. As a consequence of wt-ORF6 expression, the IKK-α stimulatory function on pIL-6 promoter activation was significantly affected (~1.2-fold decrease, *p* = 0.018; *) (Figure 3c). Taken together, these results demonstrated that the ORF6 protein does not exert its antagonistic properties, hindering NF-κB nuclear translocation but limiting its cytoplasmic activation in response to upstream stimuli.

### 4.4. NF-κB Inhibitors Counteract ORF6 Activity

**Figure 4:**
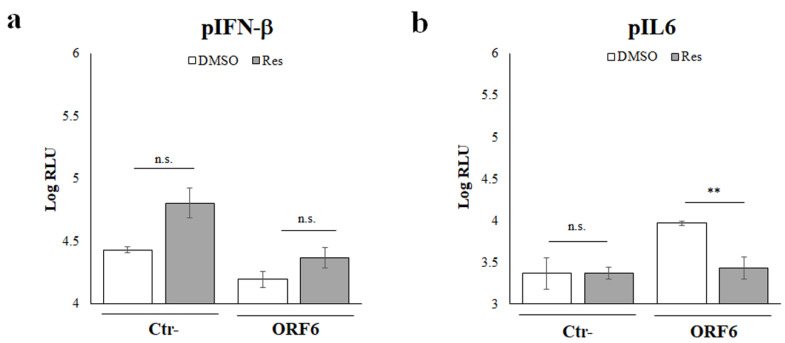
The NF-κB inhibitor *Resveratrol* is effective on ORF6 activity. HEK-293T cells were co-transfected with the IFN-β (**a**) and interleukin-6 (**b**) promoter-mediated firefly luciferase and the SV40 promoter-mediated *Renilla* luciferase reporter plasmids. Where indicated, empty plasmid (Ctr-) or ORF6 protein was ectopically expressed by transfection. At 24 h post transfection, cells were treated with 10 μM of resveratrol or with vehicle (DMSO) for additional 24 h. Firefly and *Renilla* luciferase activities were determined. Three (*n* = 3) independent experiments were performed and representative data are presented as mean logarithm values of relative luminescence unit (RLU) ± standard deviations. Significance was evaluated as *p* < 0.05 (** *p* < 0.005, , n.s. = not significant).

To further evaluate the involvement of the NF-κB transcription factor in SARS-CoV-2 ORF6 protein function, a specific inhibitor acting on the NF-κB signaling pathway was tested. A well-known group of molecules with anti-inflammatory properties is represented by polyphenols. Among them, *Resveratrol* (Res) represents the most studied and promising one [33,34]. Although its molecular mechanism is not fully characterized, some in vitro studies demonstrated *Resveratrol* activity on NF-κB activation via IκB kinase repression (IKK) [31,32,33]. In a luciferase assay, the inhibition of pIFN-β by the ectopic expression of wt-ORF6 was not significantly affected (*p* = 0.0073; **) by Res treatment compared to the relative vehicle (DMSO) treated control (Ctr-) (Figure 4a). Moreover, these results are in agreement with the marginal role of the NF-κB transcription factor on IFN-β expression.

On the contrary, we demonstrated that Res was able to revert the wt-ORF6 stimulatory effects on pIL-6 (fold change 3.4, *p* = 0.0003; ***), hindering NF-kB activation and affecting the transcriptional machinery involved in the cytokine expression (Figure 4b). Taken together, these results suggested that the SARS-CoV-2 ORF6 protein influences the NF-κB activation in the cell cytoplasm without affecting the migration of residual active transcription factor to the nucleus.

### 4.5. IL-6 and IFN-β Protein Secretion Is Affected by SARS-CoV-2 ORF6 Protein

**Figure 5:**
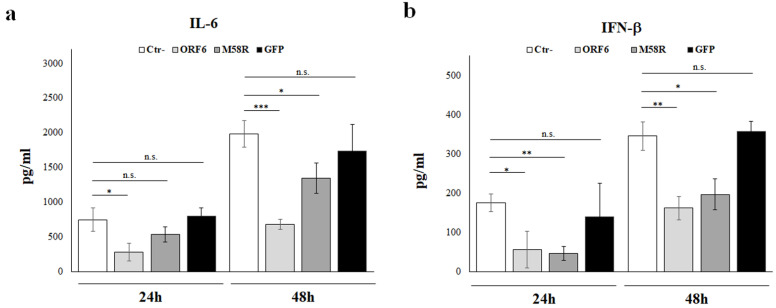
ORF6 protein hinders IL-6 secretion and IFN-β. The production of IL-6 (**a**) and IFN-β (**b**) by A549 cells was tested at early (24 h) and late (48 h) post ORF6, M58R or GFP expression by enzyme-linked immunoassay (ELISA). Negative control (Ctr-) was represented by A549 cells transfected with empty plasmid alone. Quantitative evaluation was performed based on relative standard curves and results are reported as mean concentration (pg/mL) ± standard deviations from three separate experiments (*n* = 3). Significance was evaluated as *p* < 0.05 (*** *p* < 0.0005, ** *p* < 0.005, * *p* < 0.05, n.s. = not significant).

Our previous results showed that the ORF6 retains the antagonistic nature on type I interferons. Notwithstanding, attention must be addressed to experimental data analysis and, whether possible, test viral protein activity by multiple techniques. Indeed, ORF6 displayed IFN-β antagonistic properties and, conversely, IL-6 upregulation when raw data were analyzed, but not when fold changes were calculated. For this reason, the secretion of IFN-β and IL-6 cytokines by cultured A549 cells was further evaluated by quantitative ELISA. Cell culture supernatants were collected from cells transfected with empty vector control, wt- or M58R-mutated ORF6, and green-fluorescent protein (GFP). The quantitative assessment of IFN-β and IL-6 proteins revealed that the presence of the wt-ORF6 protein abrogated both IL-6 and IFN-β release. A time-dependent depletion in IL-6 secretion was observed when the wt-ORF6 protein was over-expressed, with a 2.7- (*p* = 0.018, *) and 2.9-fold (*p* = 0.004, ***) decrease at 24 h and 48 h, respectively (Figure 5a).

In a similar manner, the presence of the viral protein led to a fold decrease of 3 (*p* = 0.01, *) and 2 (*p* = 0.002, **) at 24 h and 48 h, respectively, in IFN-β secretion (Figure 5b). Conversely, the M58R ORF6 mutant demonstrated a reduced inhibitory activity (1.5-fold decrease, *p* = 0.01; *) towards IL-6 production at 48 h, but not at 24 h post-transfection (Figure 5a). Thus, it was of interest to confirm the ORF6 inhibitory function by using alternative methods, since apparently discordant results were collected. According to reporter gene assay, IFN-β was equally downregulated by both ORF6 variants either at 24 h and 48 h post-transfection (3- and 2-fold decrease at, respectively) (Figure 5b).

### 4.6. ORF6 Protein Downregulate Cytokines Expression by Specific mRNA Nuclear Retention

**Figure 6:**
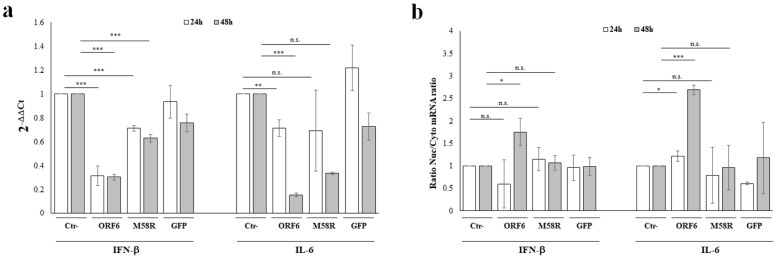
ORF6 blocks mRNA nuclear export along with transcriptional inhibition. (**a**) The effects at transcriptional level of ORF6 on IFN-β and IL-6 expression were evaluated in A549 cells. Total RNA was purified from empty vector (Ctr-), green-fluorescent protein (GFP), ORF6, or its M58R mutant transfected cells collected at both 24 h and 48 h post-transfection. Specific IFN-β and IL6 mRNA content was detected by quantitative reverse-transcription polymerase chain reaction (RT-qPCR). RNaseP gene expression was used for relative quantification based on 2^−^^ΔΔCt^ method. (**b**) Total RNA was purified from A549 nuclear and cytoplasmic cellular fractions from negative control (Ctr-), green-fluorescent protein (GFP), ORF6 or its mutant M58R expressing cells, collected at both 24 h and 48 h post-transfection. Relative quantification of IFN-β and IL6 mRNAs was performed by 2^−^^ΔΔCt^ method and the ratio between nuclear and cytoplasmic specific mRNAs content was used as a comparative method. (**a**,**b**) Three (*n* = 3) independent experiments were performed and representative data are presented as mean values ± standard deviations. Significance was evaluated as *p* < 0.05 (*** *p* < 0.0005, ** *p* < 0.005, * *p* < 0.05, n.s. = not significant).

The NF-κB major function is the transcriptional regulation of some proinflammatory cytokines expression, including IL-6. Since our previous data suggested that the NF-κB activity was not impaired by the presence of ORF6, we hypothesized other mechanisms involved in inhibiting the cytokine expression. Taking advantage of our and other results showing the ORF6 nuclear localization, we investigated its effects on IFN-β and IL-6 specific messenger RNA traffic through the nucleopore complex (NPC). A549 cells were transfected with empty vector or with the wt- or M58R mutated viral genes for 24 h or 48 h, then specific transcripts were quantitated by RT-qPCR. As additional control, samples where the ORF6 was replaced by the GFP protein were included and similarly analyzed. When cytokines expression was examined, the wt-ORF6 protein significantly downregulated endogenous IFN-β expression in transfected cells at both collecting times. As reported in Figure 6a, a 3-fold decrease was observed for IFN-β expression (*p* ≤ 0.0001; ***), without significant time-dependent variations. A similar activity, although less pronounced, was reported also for the M58R mutant (~1.4-fold decrease, *p* < 0.0001; ***) (Figure 6a).

On the other hand, a marginal effect on IL-6 transcription was observed following early wt-ORF6 ectopic expression (1.4-fold decrease, *p* = 0.002; **), while at 48 h post-transfection this effect was more pronounced, with 6-fold decrease (*p* < 0.0001, ***) in specific mRNA detection (Figure 6a). The specific effects of the ORF6 protein on the cytokine expression was proved by the evidence that minimal or no significant variations in early gene transcription occurred when either the M58R mutant or the GFP were expressed (Figure 6a). Despite the transcriptional inhibition of IFN-β and IL-6 by the wt-ORF6 protein, we additionally investigated whether the ORF6 hindered movement from the nucleus to the cytoplasm on cytokine transcripts, in order to explain the discrepancies in reporter assay results regarding IL-6. Total RNA was purified from nuclear and cytoplasmic cellular fractions, then specific mRNA content was determined by RT-qPCR in both subcellular compartments. The nuclear/cytoplasmic mRNA ratio was used as measure of judgment. At 24 h post-transfection, the nuclear retention of IFN-β mRNA was not affected by the presence of the wild-type viral protein (Figure 6b). Surprisingly, IFN-β mRNA transport was instead perturbed at 48 h post-transfection by the wt-ORF6, leading to a retention at the nuclear level of specific transcripts (fold increase 1.7, *p* = 0.01; *) (Figure 6b). On the contrary, IFN-β mRNA movement was not affected by the mutant variant or GFP (Figure 6b). Instead, IL-6 mRNA distribution within the cell compartments suggested its altered transition from the nucleus to the cytoplasm. Indeed, as early as 24 h post ORF6 expression, a 1.3-fold nuclear accumulation in IL-6 mRNA was observed (*p* = 0.02; *) (Figure 6b). This evidence was more prominent at 48 h post-transfection, when the wt-ORF6 protein accumulated in the cells, leading to a 2.7-fold nuclear retention of IL-6 mRNA (*p* < 0.0001; ***) (Figure 6b). A similar activity was not observed when the wild-type viral protein was replaced by the M58R mutant or by the GFP protein. Indeed, no significant variations in nuclear retention for IL-6 mRNAs were observed, neither at early nor late stages of protein expression (Figure 6b).

## 5. Discussion

In this study, we reported a new potential activity of the SARS-CoV-2 ORF6 protein. Although many researchers have described marked type I interferon antagonistic properties for the selected viral protein [25,26], we described a new strategy that could be used by the ORF6 viral protein to counteract host cell response and cellular processes in order to favor virus replication and spread. The standard method used to measure the activation rate of any promoter is the luciferase reporter assay after normalization with the internal control *Renilla* luciferase [35,36]. In the present study, we demonstrated that the SARS-CoV-2 ORF6 expression determined changes in the specific promoters’ activation herein investigated. Notwithstanding, the specific antagonistic properties of the ORF6 protein was always associated with a strong decrease in the *Renilla* luciferase internal control, even if it was expressed at nearly undetectable levels, suggesting that the viral protein effects non-specific perturbations of cellular activities. This new evidence corroborated our hypothesis that a broad-range host translation inhibition by the viral protein occurred, although not directly investigated. For this reason, luciferase data normalization (by firefly/*Renilla* ratio) consistently affected evaluation of the results and was not applicable. Thus, firefly luciferase raw data, expressed as relative light units (RLU), were used in order to evaluate if the ORF6 specific antagonistic function. Moreover, the ORF6 M58R mutant, carrying a point mutation at the C-terminus which is critical for some protein functions, did not display a similar activity. The antagonistic property of the wild-type ORF6 viral protein on full-length IFN-β promoter was confirmed. Instead, the loss of activity of the M58R mutant provided new insight into the understanding of SARS-CoV-2 counteracting strategies on host defenses. Notwithstanding, further analysis of the ORF6 function on promoter activation of cytokines other than IFN-β were conducted in an attempt to better address its molecular mechanism. In this context, we reported that the viral protein mostly targeted interleukin-6 (IL-6), which represents one of the most important cytokines involved in the so-called cytokine storm occurring during viral infection [19,20,21,37,38,39]. On the contrary, loss of activity on interleukin-10 (IL-10) supported the evidence that the SARS-CoV-2 ORF6 protein was not involved in the control of counter-regulatory cytokines, such as IL-10, that suppresses the production of pro-inflammatory cytokines [40,41,42]. Indeed, while moderate antagonistic properties were detected with respect to IL-10 promoter activation, IL-6 promoter resulted upregulated by the ORF6, which—in turn—did not match with IL-6 protein secretion. The observation suggested the presence of multiple factors involved in controlling the inflammatory response by the ORF6 viral protein [25,26,27,28,29,30,31,32]. On the contrary, the antagonistic activity of the ORF6 protein was appreciated towards IFN-β and IL-6 minimal promoter, responding only to the effect of the NF-κB transcription factor. NF-κB is a ubiquitous transcription factor whose activation and nuclear translocation, along with the control of several cellular processes, result in the expression of pro-inflammatory genes (IL-6, TNF-α, and IL-1β) [43,44,45,46,47]. Although NF-κB is known to function as an activator of transcription, its interaction with other activated transcription factors is fundamental to determine the transcription rates of NF-κB-dependent cytokine genes. For example, cooperative binding with the transcription factor NF–IL-6 is required for the transcriptional activation of both IL-8 and IL-6 [47,48]. On the contrary, NF-κB involvement in controlling IFNs expression is still unclear and is supposed to be marginal, unlike IRF-3/7 transcription factors [49,50,51]. This is in agreement with the evidence that the ORF6 retained antagonistic function on IFN-β promoter activation and its endogenous transcription, since factors other than NF-κB are targeted by the ORF6 protein—such as the RIG-I-MAVS-IRF-3 signaling pathway—in order to counteract the host innate immune response [25]. In parallel, we demonstrated that the ORF6 protein, despite being associated with Rae1-Nup98 complex [27,28,29,30], did not hinder NF-κB nuclear translocation, contrary to that which has been observed for other transcription factors, such as STAT-1/2. This evidence may explain the increased IL-6 transcriptional activity determined by reporter gene assay when the ORF6 was ectopically expressed [27]. Moreover, NF-κB involvement in ORF6 function was further supported by the evidence that the IκB kinase (IKK)—an activator of NF-κB—activity was similarly repressed by the viral protein. Therefore, the ORF6 protein prevents NF-κB activation in the cell cytoplasm rather than hampering its nuclear translocation. Additionally, treatment with the IKK inhibitor *Resveratrol* confirmed that this transcription factor is directly involved in the ORF6 function [33,52,53,54]. Surprisingly, neither IFN-β nor IL-6 proteins were detected in cell culture supernatants of A549 cells expressing ORF6. These findings were consistent with the possibility that ORF6 could be a double-edged sword exerting a non-specific perturbation of cellular activities, such as general transcription, but also modulating mRNAs translation. Thus, an alternative mechanism of action for ORF6 was supposed to control the expression of IL-6. A plausible alternative mechanism was issued by RT-qPCR of messenger RNAs for IL-6. A minor reduction in IL-6 mRNA following early expression of the viral protein—i.e., within 24 h—was noted, while a marked reduction was evident later on, as hindrance of general transcription or widespread degradation of host cytoplasmic basal mRNAs. Notwithstanding, we established that ORF6 exerted a significant influence on mRNA transition from the nucleus to the cytoplasm since the vast majority of mRNAs remained trapped at a nuclear level when the wild-type ORF6—but not its C-terminal mutant—was expressed. Consequently, only a small amount of these were found in the cytoplasm, and thus available for protein translation. This newly described ORF6 activity was further corroborated by a similar phenomenon with respect to IFN-β, although it was already blocked at transcriptional level. A similar mechanism has been already described for several viruses, including Influenza A virus, Zika virus, and other viruses in order to overcome host response [55,56]. This explains why ORF6 exhibits antagonistic activity against a variety of cellular mediators, including inflammatory cytokines, whose downregulation has been observed at both protein and transcriptional level due to an increased nuclear retention when the ORF6 protein was expressed. To some extent, this activity has been described for SARS-CoV-2 [27,28,29,30], but the specific blockage on cytokine mRNAs by the viral protein ORF6 was not yet demonstrated. Thus, ORF6-mediated inhibition of host mRNA export allows the SARS-CoV-2 virus to translate its mRNAs in the cytoplasm while avoiding competition with host mRNAs for the translation machinery, including antiviral factors such as interferons and IL-6. Unavoidably, a similar activity leads to the shutdown of several cellular physiological activities and cytotoxic consequences which, on the other hand, are favorable for virus replication and spread [29,30]. On the contrary, viral components other than the ORF6 protein could underlie the exacerbated IL-6 production described in the SARS-CoV-2 infection, which occurred in an uncontrolled leading to tissue damage rather than an appropriate host defense [57]. Moreover, we demonstrated that the antagonistic effects on IFN-β and IL-6 of SARS-CoV-2 ORF6 protein were strikingly influenced by the domain located at the C-terminus of the protein. Indeed, the M58R mutant inhibitory activity towards the selected cytokines was significantly attenuated and we speculated that this behavior is a consequence of the loss in disrupting the nuclear mRNA export processes [27,28,29,30,58].

The highly pathogenic effects of ORF6 were also demonstrated in vivo. Zhu et al. reported early lethality, reduced trachea branching, and muscle injury in flies over-expressing the viral protein, but treatment with the NF-κB inhibitor selinexor attenuated the deleterious effects caused by ORF6 reducing inflammatory cytokines release, including IL-6 [58]. This new knowledge regarding SARS-CoV-2 pathogenesis opens the way to new therapeutic strategies, as the viral protein ORF6 seems to be one of the most important virulence factors acting as a multifunctional modulator of host cellular processes, including antiviral ones.

## 6. Conclusions

In the present study, we described a new potential mechanism for an efficient IFN-β suppression by the SARS-CoV-2 ORF6 protein, which involves the inactivation of nucleopore complex proteins (NPC) involved in mRNA movement from nucleus to cytoplasm in order to be translated. Despite the ORF6 inhibitory function on NPC also being responsible for the suppression of inflammatory mediators such as IL-6, it did not impair NF-κB nuclear translocation; although its cytoplasmic activation was restricted by the viral protein. In the light of these new findings, we speculated that the ORF6 inhibitory activity towards nucleopore traffic would be a potential target for a specific therapy. In particular, broad-spectrum anti-inflammatory and selective inhibitors of nuclear export (SINE) compounds—such as selinexor—which elicit the activation of several anti-inflammatory, antioxidant, and cytoprotective transcription factors (IκB, Nrf2) may be useful in controlling COVID-19 symptoms [59,60,61,62,63]. Moreover, since naturally occurring ORF6 mutants are frequently reported, the effects of these mutations should be considered to better understand viral escape from the immune system. In addition, the development of an elevated antibody response to the ORF6 protein elicited in infected children suggests that the viral protein is an important factor in driving control of SARS-CoV-2 infection [64,65].

## Data Availability

Not applicable.

## Supplementary Materials

The following supporting information can be downloaded at: https://www.mdpi.com/article/10.3390/v14061273/s1, Raw dataset, original figures.

## References

[1] Cui J., Li F., Shi Z.L. (2019). Origin and evolution of pathogenic coronaviruses. Nat. Rev. Microbiol..

[2] Zhu N., Zhang D., Wang W., Li X., Yang B., Song J., Zhao X., Huang B., Shi W., Lu R. (2020). A Novel Coronavirus from Patients with Pneumonia in China, 2019. N. Engl. J. Med..

[3] World Health Organization (2020). Coronavirus Disease 2019 (COVID-19): Situation Summary.

[4] Chen Y., Liu Q., Guo D. (2020). Emerging coronaviruses: Genome structure, replication, and pathogenesis. J. Med. Virol..

[5] Guan W.J., Ni Z.Y., Hu Y., Liang W.H., Ou C.Q., He J.X., Liu L., Shan H., Lei C.L., Hui D.S.C. (2020). Clinical Characteristics of Coronavirus Disease 2019 in China. N. Engl. J. Med..

[6] Huang C., Wang Y., Li X., Ren L., Zhao J., Hu Y., Zhang L., Fan G., Xu J., Gu X. (2020). Clinical features of patients infected with 2019 novel coronavirus in Wuhan, China. Lancet.

[7] Wang D., Hu B., Hu C., Zhu F., Liu X., Zhang J., Wang B., Xiang H., Cheng Z., Xiong Y. (2020). Clinical Characteristics of 138 Hospitalized Patients with 2019 Novel Coronavirus-Infected Pneumonia in Wuhan, China. JAMA.

[8] Hui K.P.Y., Cheung M.C., Perera R.A.P.M., Ng K.C., Bui C.H.T., Ho J.C.W., Ng M.M.T., Kuok D.I.T., Shih K.C., Tsao S.W. (2020). Tropism, replication competence, and innate immune responses of the coronavirus SARS-CoV-2 in human respiratory tract and conjunctiva: An analysis in ex-vivo and in-vitro cultures. Lancet Respir. Med..

[9] Zou L., Ruan F., Huang M., Liang L., Huang H., Hong Z., Yu J., Kang M., Song Y., Xia J. (2020). SARS-CoV-2 Viral Load in Upper Respiratory Specimens of Infected Patients. N. Engl. J. Med..

[10] Davidson A.D., Williamson M.K., Lewis S., Shoemark D., Carroll M.W., Heesom K.J., Zambon M., Ellis J., Lewis P.A., Hiscox J.A. (2020). Characterisation of the transcriptome and proteome of SARS-CoV-2 reveals a cell passage induced in-frame deletion of the furin-like cleavage site from the spike glycoprotein. Genome Med..

[11] Hadjadj J., Yatim N., Barnabei L., Corneau A., Boussier J., Smith N., Péré H., Charbit B., Bondet V., Chenevier-Gobeaux C. (2020). Impaired type I interferon activity and inflammatory responses in severe COVID-19 patients. Science.

[12] Reikine S., Nguyen J.B., Modis Y. (2014). Pattern Recognition and Signaling Mechanisms of RIG-I and MDA5. Front. Immunol..

[13] Berke I.C., Li Y., Modis Y. (2013). Structural basis of innate immune recognition of viral RNA. Cell Microbiol..

[14] Libermann T.A., Baltimore D. (1990). Activation of interleukin-6 gene expression through the NF-kappa B transcription factor. Mol. Cell. Biol..

[15] Blanco-Melo D., Nilsson-Payant B.E., Liu W.C., Uhl S., Hoagland D., Møller R., Jordan T.X., Oishi K., Panis M., Sachs D. (2020). Imbalanced Host Response to SARS-CoV-2 Drives Development of COVID-19. Cell.

[16] Lei X., Dong X., Ma R., Wang W., Xiao X., Tian Z., Wang C., Wang Y., Li L., Ren L. (2020). Activation and evasion of type I interferon responses by SARS-CoV-2. Nat. Commun..

[17] Acharya D., Liu G., Gack M.U. (2020). Dysregulation of type I interferon responses in COVID-19. Nat. Rev. Immunol..

[18] Luo Y., Zheng S.G. (2016). Hall of Fame among Pro-inflammatory Cytokines: Interleukin-6 Gene and Its Transcriptional Regulation Mechanisms. Front. Immunol..

[19] Karin M., Greten F.R. (2005). NF-kappaB: Linking inflammation and immunity to cancer development and progression. Nat. Rev. Immunol..

[20] Neurath M.F., Becker C., Barbulescu K. (1998). Role of NF-kappaB in immune and inflammatory responses in the gut. Gut.

[21] Oh S.J., Shin O.S. (2021). SARS-CoV-2 Nucleocapsid Protein Targets RIG-I-Like Receptor Pathways to Inhibit the Induction of Interferon Response. Cells.

[22] Gori Savellini G., Anichini G., Gandolfo C., Cusi M.G. (2021). SARS-CoV-2 N Protein Targets TRIM25-Mediated RIG-I Activation to Suppress Innate Immunity. Viruses.

[23] Hu Y., Li W., Gao T., Cui Y., Jin Y., Li P., Ma Q., Liu X., Cao C. (2017). The Severe Acute Respiratory Syndrome Coronavirus Nucleocapsid Inhibits Type I Interferon Production by Interfering with TRIM25-Mediated RIG-I Ubiquitination. J. Virol..

[24] Chen K., Xiao F., Hu D., Ge W., Tian M., Wang W., Pan P., Wu K., Wu J. (2020). SARS-CoV-2 Nucleocapsid Protein Interacts with RIG-I and Represses RIG-Mediated IFN-β Production. Viruses.

[25] Shemesh M., Aktepe T.E., Deerain J.M., McAuley J.L., Audsley M.D., David C.T., Purcell D., Urin V., Hartmann R., Moseley G.W. (2021). SARS-CoV-2 suppresses IFNβ production mediated by NSP1, 5, 6, 15, ORF6 and ORF7b but does not suppress the effects of added interferon. PLoS Pathog..

[26] Yuen C.K., Lam J.Y., Wong W.M., Mak L.F., Wang X., Chu H., Cai J.P., Jin D.Y., To K.K., Chan J.F. (2020). SARS-CoV-2 nsp13, nsp14, nsp15 and orf6 function as potent interferon antagonists. Emerg. Microbes Infect..

[27] Miorin L., Kehrer T., Sanchez-Aparicio M.T., Zhang K., Cohen P., Patel R.S., Cupic A., Makio T., Mei M., Moreno E. (2020). SARS-CoV-2 Orf6 hijacks Nup98 to block STAT nuclear import and antagonize interferon signaling. Proc. Natl. Acad. Sci. USA.

[28] Addetia A., Lieberman N.A.P., Phung Q., Hsiang T.Y., Xie H., Roychoudhury P., Shrestha L., Loprieno M.A., Huang M.L., Gale M. (2021). SARS-CoV-2 ORF6 Disrupts Bidirectional Nucleocytoplasmic Transport through Interactions with Rae1 and Nup98. MBio.

[29] Lee J.G., Huang W., Lee H., van de Leemput J., Kane M.A., Han Z. (2021). Characterization of SARS-CoV-2 proteins reveals Orf6 pathogenicity, subcellular localization, host interactions and attenuation by Selinexor. Cell Biosci..

[30] Hall R., Guedán A., Yap M.W., Young R.G., Harvey R., Stoye J.P., Bishop K.N. (2022). SARS-CoV-2 ORF6 disrupts innate immune signalling by inhibiting cellular mRNA export. bioRxiv.

[31] Burke J.M., St Clair L.A., Perera R., Parker R. (2021). SARS-CoV-2 infection triggers widespread host mRNA decay leading to an mRNA export block. RNA.

[32] Kimura I., Konno Y., Uriu K., Hopfensperger K., Sauter D., Nakagawa S., Sato K. (2021). Sarbecovirus ORF6 proteins hamper induction of interferon signaling. Cell Rep..

[33] Ma C., Wang Y., Dong L., Li M., Cai W. (2015). Anti-inflammatory effect of resveratrol through the suppression of NF-κB and JAK/STAT signaling pathways. Acta Biochim. Biophys. Sin..

[34] De Sá Coutinho D., Pacheco M.T., Frozza R.L., Bernardi A. (2018). Anti-Inflammatory Effects of Resveratrol: Mechanistic Insights. Int. J. Mol. Sci..

[35] Nakajima Y., Kimura T., Sugata K., Enomoto T., Asakawa A., Kubota H., Ikeda M., Ohmiya Y. (2005). Multicolor luciferase assay system: One-step monitoring of multiple gene expressions with a single substrate. Biotechniques.

[36] Gong D.W., Bi S., Pratley R.E., Weintraub B.D. (1996). Genomic structure and promoter analysis of the human obese gene. J. Biol. Chem..

[37] Hu B., Huang S., Yin L. (2021). The cytokine storm and COVID-19. J. Med. Virol..

[38] Fajgenbaum D.C., June C.H. (2020). Cytokine Storm. N. Engl. J. Med..

[39] Gao Y.M., Xu G., Wang B., Liu B.C. (2021). Cytokine storm syndrome in coronavirus disease 2019: A narrative review. J. Intern. Med..

[40] Moore K.W., de Waal Malefyt R., Coffman R.L., O'Garra A. (2001). Interleukin-10 and the interleukin-10 receptor. Annu. Rev. Immunol..

[41] Wang P., Wu P., Siegel M.I., Egan R.W., Billah M.M. (1995). Interleukin (IL)-10 inhibits nuclear factor kappa B (NF kappa B) activation in human monocytes. IL-10 and IL-4 suppress cytokine synthesis by different mechanisms. J. Biol. Chem..

[42] Raychaudhuri B., Fisher C.J., Farver C.F., Malur A., Drazba J., Kavuru M.S., Thomassen M.J. (2000). Interleukin 10 (IL-10)-mediated inhibition of inflammatory cytokine production by human alveolar macrophages. Cytokine.

[43] Carcaterra M., Caruso C. (2021). Alveolar epithelial cell type II as main target of SARS-CoV-2 virus and COVID-19 development via NF-Kb pathway deregulation: A physio-pathological theory. Med. Hypotheses.

[44] Pflug K.M., Sitcheran R. (2020). Targeting NF-κB-Inducing Kinase (NIK) in Immunity.; Inflammation.; and Cancer. Int. J. Mol. Sci..

[45] Mollaei M., Abbasi A., Hassan Z.M., Pakravan N. (2020). The intrinsic and extrinsic elements regulating inflammation. Life Sci..

[46] Schow S.R., Joly A. (1997). N-acetyl-leucinyl-leucinyl-norleucinal inhibits lipopolysaccharide-induced NF-kappaB activation and prevents TNF and IL-6 synthesis in vivo. Cell Immunol..

[47] Akira S., Kishimoto T. (1992). IL-6 and NF-IL6 in acute-phase response and viral infection. Immunol. Rev..

[48] Mukaida N., Hishinuma A., Zachariae C.O., Oppenheim J.J., Matsushima K. (1991). Regulation of human interleukin 8 gene expression and binding of several other members of the intercrine family to receptors for interleukin-8. Adv. Exp. Med. Biol..

[49] Doyle S., Vaidya S., O'Connell R., Dadgostar H., Dempsey P., Wu T., Rao G., Sun R., Haberland M., Modlin R. (2002). IRF3 mediates a TLR3/TLR4-specific antiviral gene program. Immunity.

[50] Sato M., Suemori H., Hata N., Asagiri M., Ogasawara K., Nakao K., Nakaya T., Katsuki M., Noguchi S., Tanaka N. (2000). Distinct and essential roles of transcription factors IRF-3 and IRF-7 in response to viruses for IFN-alpha/beta gene induction. Immunity.

[51] Wang X., Hussain S., Wang E.J., Wang X., Li M.O., García-Sastre A., Beg A.A. (2007). Lack of essential role of NF-kappa B p50.; RelA.; and cRel subunits in virus-induced type 1 IFN expression. J. Immunol..

[52] He Y., Lu X., Chen T., Yang Y., Zheng J., Chen C., Zhang Y., Lei W. (2021). Resveratrol protects against myocardial ischemic injury via the inhibition of NF-κB-dependent inflammation and the enhancement of antioxidant defenses. Int. J. Mol. Med..

[53] Faith S.A., Sweet T.J., Bailey E., Booth T., Docherty J.J. (2006). Resveratrol suppresses nuclear factor-kappaB in herpes simplex virus infected cells. Antiviral. Res..

[54] Hariharan A., Hakeem A.R., Radhakrishnan S., Reddy M.S., Rela M. (2021). The Role and Therapeutic Potential of NF-kappa-B Pathway in Severe COVID-19 Patients. Inflammopharmacology.

[55] Leon K., Flynn R., Khalid M.M., Fontaine K.A., Nguyen T., Renuka Kumar G., Simoneau C.R., Tomar S., Jimenez-Morales D., Dunlap M. (2020). Zika Virus Infection Prevents Host mRNA Nuclear Export by Disrupting UPF1 Function. bioRxiv.

[56] Kuss S.K., Mata M.A., Zhang L., Fontoura B.M. (2013). Nuclear imprisonment: Viral strategies to arrest host mRNA nuclear export. Viruses.

[57] Su C.M., Wang L., Yoo D. (2021). Activation of NF-κB and induction of proinflammatory cytokine expressions mediated by ORF7a protein of SARS-CoV-2. Sci. Rep..

[58] Zhu J.Y., Lee J.G., van de Leemput J., Lee H., Han Z. (2021). Functional analysis of SARS-CoV-2 proteins in Drosophila identifies Orf6-induced pathogenic effects with Selinexor as an effective treatment. Cell Biosci..

[59] Kashyap T., Argueta C., Aboukameel A., Unger T.J., Klebanov B., Mohammad R.M., Muqbil I., Azmi A.S., Drolen C., Senapedis W. (2016). Selinexor, a Selective Inhibitor of Nuclear Export (SINE) compound, acts through NF-κB deactivation and combines with proteasome inhibitors to synergistically induce tumor cell death. Oncotarget.

[60] Kashyap T., Murray J., Walker C.J., Chang H., Tamir S., Hou B., Shacham S., Kauffman M.G., Tripp R.A., Landesman Y. (2021). Selinexor, a novel selective inhibitor of nuclear export, reduces SARS-CoV-2 infection and protects the respiratory system in vivo. Antivir. Res..

[61] Tajiri N., De La Peña I., Acosta S.A., Kaneko Y., Tamir S., Landesman Y., Carlson R., Shacham S., Borlongan C.V. (2016). A Nuclear Attack on Traumatic Brain Injury: Sequestration of Cell Death in the Nucleus. CNS Neurosci. Ther..

[62] Olagnier D., Farahani E., Thyrsted J., Blay-Cadanet J., Herengt A., Idorn M., Hait A., Hernaez B., Knudsen A., Iversen M.B. (2020). SARS-CoV2-mediated suppression of NRF2-signaling reveals potent antiviral and anti-inflammatory activity of 4-octyl-itaconate and dimethyl fumarate. Nat. Commun..

[63] Shah A. (2020). Novel Coronavirus-Induced NLRP3 Inflammasome Activation: A Potential Drug Target in the Treatment of COVID-19. Front. Immunol..

[64] Hachim A., Gu H., Kavian O., Kwan M.Y., Chan W.H., Yau Y.S., Chiu S.S., Tsang O.T., Hui D.S., Ma F. (2021). The SARS-CoV-2 antibody landscape is lower in magnitude for structural proteins, diversified for accessory proteins and stable long-term in children. medRxiv.

[65] Hachim A., Kavian N., Valkenburg S.A. (2021). Antibody landscapes of SARS-CoV-2 can reveal novel vaccine and diagnostic targets. Curr. Opin. Virol..

