# Supplementary material for: Nucleopore Traffic Is Hindered by SARS-CoV-2 ORF6 Protein to Efficiently Suppress IFN-β and IL-6 Secretion
Source: Viruses. 2022 Jun 11;14(6):1273. doi: 10.3390/v14061273 (PMC9230033; doi:10.3390/v14061273)
Supplement: Supplementary file 1 [file viruses-14-01273-s001.zip › Original Images.pdf]

Nucleopore traffic is hindered by SARS-CoV-2 ORF6 protein to efficiently suppress IFN-β and IL-6 secretion

Gianni Gori Savellini [1*], Gabriele Anichini [1], Claudia Gandolfo [1], Chiara Terrosi [1], Maria Grazia Cusi

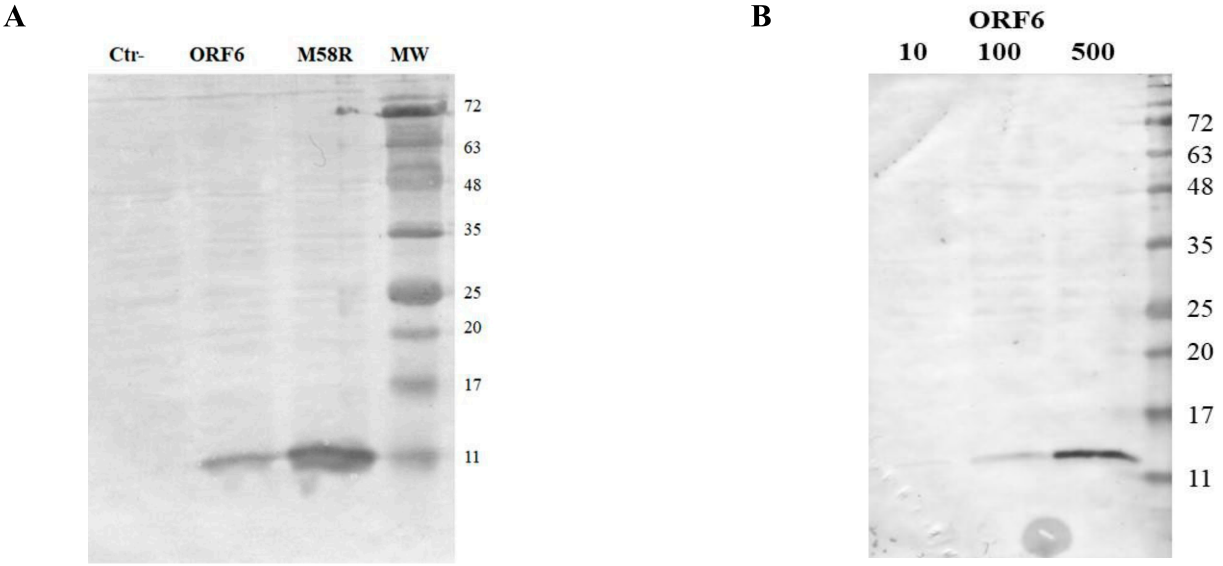

Figure S1. **Inhibitory activity of SARS-COV-2 ORF6 protein.**

HEK-293T cells were co-transfected with the IFN-β promoter-mediated Firefly luciferase (pIFN-β) and the SV40 promoter-mediated *Renilla* luciferase reporter plasmids, with (A) ORF6 or the mutant M58R expressing plasmids or with empty vector (Ctr-) or (B) increasing amounts of ORF6 expressing plasmid. At 48h post-transfection, cells were collected for reporter assay and 25 μg of total proteins were resolved by SDS-PAGE followed by immunostaining with anti-HA monoclonal antibody to confirm ORF6 protein variants expression. MW: molecular weight. The immunoblotting image is representative of experiments performed at least three times.

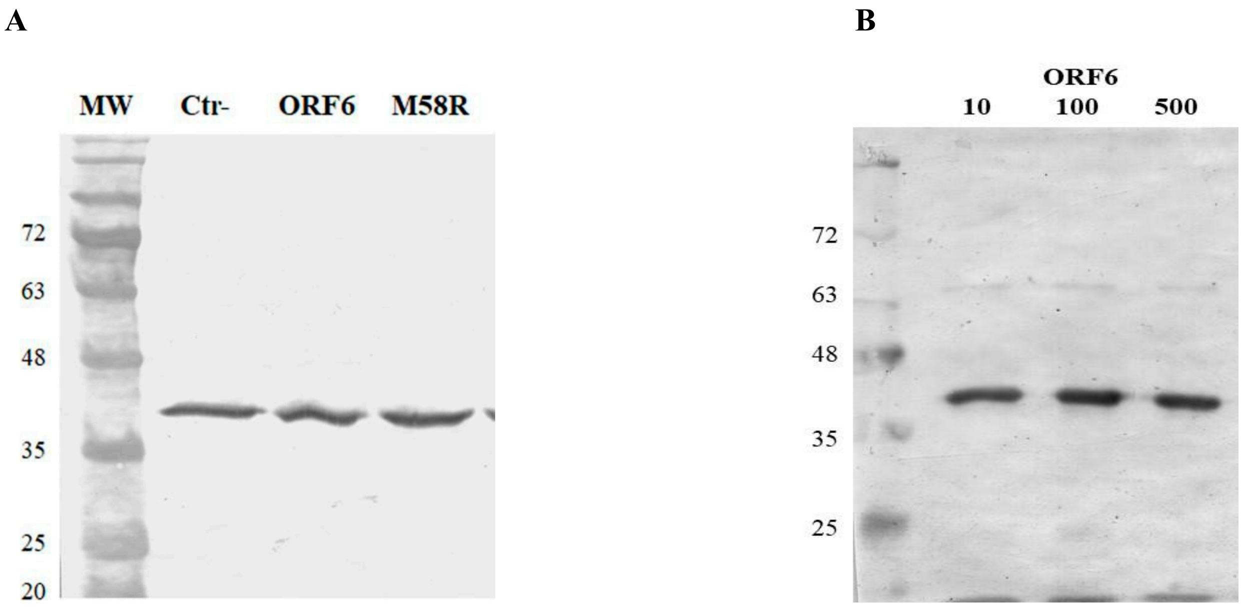

Figure S1a. **Inhibitory activity of SARS-COV-2 ORF6 protein.**

HEK-293T cells were co-transfected with the IFN-β promoter-mediated Firefly luciferase (pIFN-β) and the SV40 promoter-mediated *Renilla* luciferase reporter plasmids, with (A) ORF6 or the mutant M58R expressing plasmids or with empty vector (Ctr-) and (B) with increasing amounts of ORF6 expressing plasmid. At 48h post-transfection, cells were collected for reporter assay and 25 μg of total proteins were resolved by SDS-PAGE followed by immunostaining with anti-actin monoclonal antibody as loading control. MW: molecular weight. The immunoblotting image is representative of experiments performed at least three times.

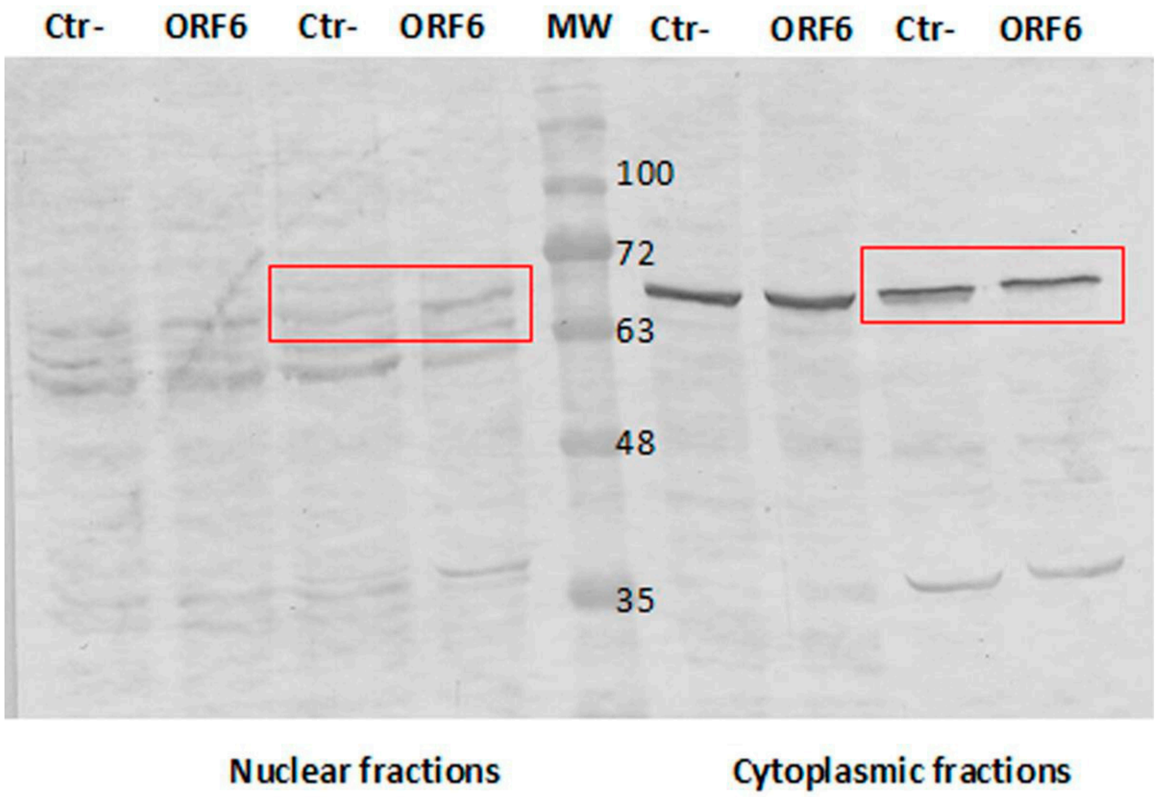

Figure S3b. NF-κB activity is affected by ORF6 protein activity.

NF-κB nuclear translocation was evaluated in empty vector (Ctr-) or ORF6 expressing A549 cells. Cellular fractionation was performed as described in the material and methods section and equal amounts (25 μg) of proteins were resolved by denaturing polyacrylamide gel. NF-κB movement into the nucleus (left side) or retained in the cell cytoplasm (right side) was evaluated by probing the samples with a specific antibody. MW: molecular weight. The immunoblotting image is representative of experiments performed at least three times.

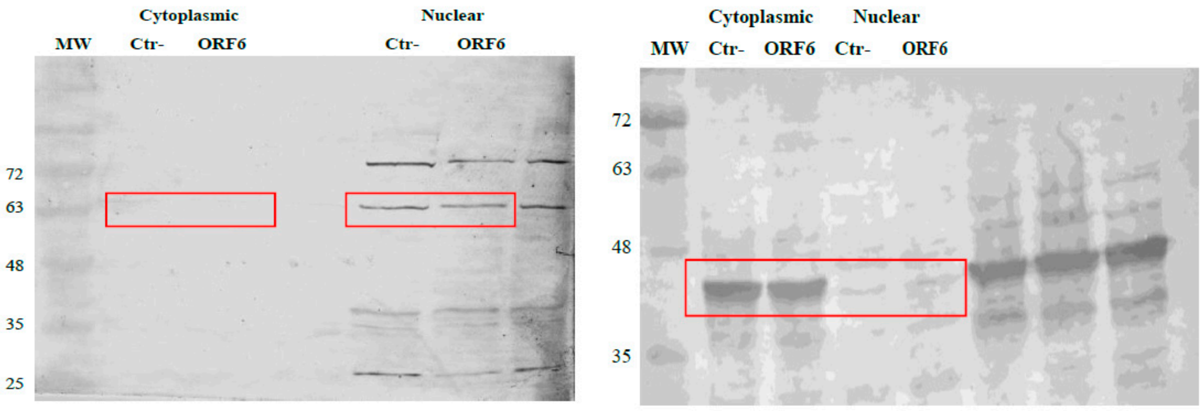

Figure S3b. NF-κB activity is affected by ORF6 protein activity.

Cellular fractionation was performed on empty vector (Ctr-) or ORF6 expressing A549 cells as described in the material and methods section. Equal amounts of nuclear or cytoplasmatic proteins (25 µg) were resolved by denaturing polyacrylamide gel and immunoblotting for Lamin B1 (left panel) or actin (right panel) detection was performed to validate the experimental procedure. MW: molecular weight. The reported image is representative of at least three experiments.

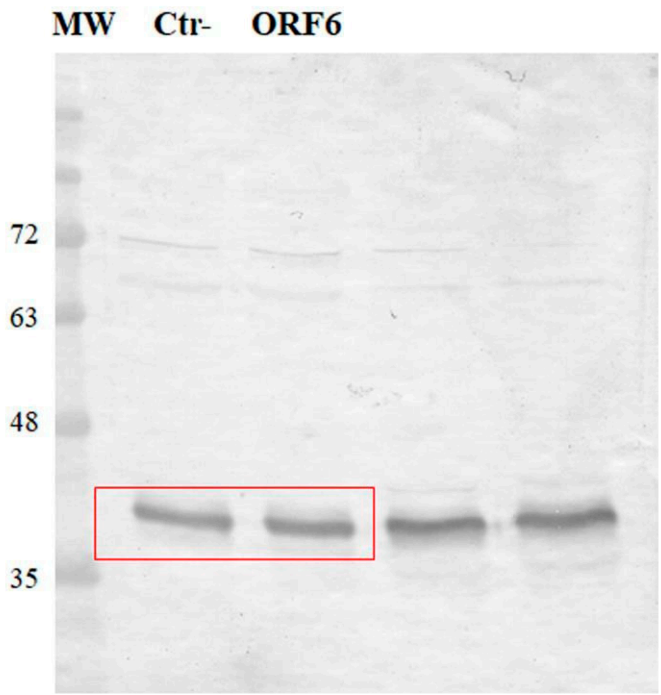

Figure S3b. NF-κB activity is affected by ORF6 protein activity.

Cellular fractionation was performed on empty vector (Ctr-) or ORF6 expressing A549 cells as described in the material and methods section. Equal amounts of cytoplasmic proteins (25 μg) were resolved by denaturing polyacrylamide gel and actin was probed as loading control for further experiments. MW: molecular weight. The reported image is representative of at least three experiments.

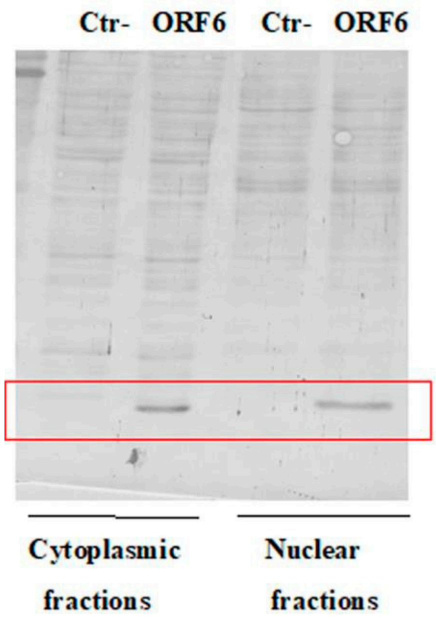

Figure S3b. NF-κB activity is affected by ORF6 protein activity.

Cellular fractionation was performed on empty vector (Ctr-) or ORF6 expressing A549 cells as described in the material and methods section. Equal amounts of cytoplasmic or nuclear proteins (25 µg) were resolved by denaturing polyacrylamide gel and ORF6 subcellular localization was assessed by anti-HA epitope tag antibody staining. MW: molecular weight. The reported image is representative of at least three experiments.
